# Japan’s development cooperation for health in Vietnam: a first holistic assessment on Japan’s ODA and non-ODA public resources cooperation

**DOI:** 10.1186/s12889-021-12170-0

**Published:** 2021-11-27

**Authors:** Sangnim Lee, Aya Ishizuka, Hisateru Tachimori, Manami Uechi, Hidechika Akashi, Eiji Hinoshita, Hiroaki Miyata, Kenji Shibuya

**Affiliations:** 1 Institute for Global Health Policy Research, Bureau of International Health Cooperation, National Center for Global Health and Medicine, 1-21-1 Toyama, Shinjuku-ku, Tokyo, 162-8655 Japan; 2 Disease Control and Prevention Center, National Center for Global Health and Medicine, Tokyo, Japan; 3 Department of Epidemiology and Clinical Research, the Research Institute of Tuberculosis, Japan Anti-Tuberculosis Association, Tokyo, Japan; 4 Department of Global Health Policy, Graduate School of Medicine, The University of Tokyo, Tokyo, Japan; 5 Endowed Course for Health System Innovation, Keio University School of Medicine, Tokyo, Japan; 6 Center for Global Health, Massachusetts General Hospital, Boston, MA United States of America; 7 Bureau of International Health Cooperation, National Center for Global Health and Medicine, Tokyo, Japan; 8 Health and Medical Division, Bureau of Personnel and Education, Ministry of Defense, Tokyo, Japan; 9 Department of Health Policy and Management, Keio University School of Medicine, Tokyo, Japan; 10 Soma COVID Vaccination Medical Center, Fukushima, Japan

**Keywords:** Japan, ODA, Health policy, Vietnam, UHC, Health system strengthening, Health systems, Development cooperation, Development assistance for health, Project monitoring and evaluation

## Abstract

**Background:**

Japan strives to strengthen its development cooperation by mobilizing various resources to assist partner countries advance on Universal Health Coverage by 2030. However, the involvement and roles of various actors for health are not clear. This study is the first to map Japan’s publicly funded projects by both Official Development Assistance (ODA) and other non-ODA public funds, and to describe the intervention areas. Further, the policy implications for country-specific cooperation strategies are discussed. The development cooperation for health in Vietnam is used as a case in this study.

**Methods:**

A cross-sectional analysis of the Japanese publicly funded health projects that were being implemented in Vietnam during December 2016 was conducted. A framework of analysis based on the World Health Organization six health systems building blocks was adopted. The projects’ qualitative information was also assessed.

**Results:**

Overall, 68 projects implemented through Japanese public funding were analyzed. These 68 projects under 15 types of schemes were managed by seven different scheme-operating organizations and funded by five ministries. Of these 44 (64.7%) were ODA and 24 (35.3%) were non-ODA projects. Among the recategorized six building blocks of the health system, the largest proportion of projects was health service delivery (44%), followed by health workforces (25%), and health information systems (15%). Almost half the projects were implemented together with the central hospitals as Vietnamese counterparts, which suggests that this is one area in which the specificities of Japanese cooperation are demonstrated. No synergetic effects of potential collaboration or harmonization among Japanese funded projects were captured.

**Conclusions:**

Several Japanese-funded projects addressed a wide range of health issues across all six building blocks of the health system in Vietnam. However, there is room for improvement in developing coordination and harmonization among the diversified Japanese projects. Establishing a country-specific mechanism for strategic coordination across Japanese ministries’ schemes can yield efficient and effective development cooperation for health. While Vietnam’s dependence on external funding is low, the importance of coordination across domestic actors of the donor countries can serve as an important lesson, especially in beneficiary countries with high external funding dependency.

**Supplementary Information:**

The online version contains supplementary material available at 10.1186/s12889-021-12170-0.

## Background

Universal health coverage (UHC) of quality and affordable essential health services for all is the core driver of health sustainable development goals (SDGs) [1]. For the world to achieve UHC by 2030, various resources need to be mobilized at the national and global levels [1, 2]. Japan was the fourth largest donor of disbursements in Official Development Assistance (ODA) in 2019 [3]. Projects operated under the ODA funds are strategized and systematically managed under its funding ministry, the Ministry of Foreign Affairs (MOFA), and the implementation agency, Japan International Cooperation Agency (JICA).

Since Japan’s “ODA Charter” was approved by the Cabinet in 1992 and revised in 2003, it has become the basis of Japan’s ODA policy. It was further revised in 2015, and the name was changed to “the Development Cooperation Charter,” based on the recognition of recent trends of international communities’ efforts to address global challenges [4]. It emphasizes the promotion of development cooperation by not only mobilizing ODA, but also by collaborating with other funding resources and activities of the government, and various entities such as private sector corporations and civil society organizations. The charter aims to achieve peace, stability, and prosperity of the international community, which, it explains, will also be beneficial to Japan. In addition, a health sector specific policy of the Charter called “the Basic Design for Peace and Health” was approved by the Headquarters for Healthcare Policy headed by the prime minister and adopted in the same year [5]. This policy highlights the importance of health systems strengthening (HSS) in order to achieve UHC and illustrates the government’s plan to promote development cooperation for health by mobilizing Japan’s expertise, experience, technology, and medical products through the utilization of various public resources [5].

These ODA policies align with Japan’s domestic health policies as well. In 2014, the government formulated the Healthcare Policy under the newly passed Act to Promote Healthcare and Medical Strategy [6], which aims to promote health and longevity in Japan as well as abroad. The Act is grounded on the idea of mutual growth and seeks to facilitate the overseas expansion of Japan’s medical products, technologies, and services.

The development of the ODA and domestic health policies has promoted both development cooperation for health as well as the overseas expansion of the Japanese health sector. As a result, Japan’s development cooperation for health in recipient countries has been implemented by not only ODA-related agencies, but also by other entities such as the Ministry of Health, Labour and Welfare (MHLW), the Ministry of Economy, Trade, and Industry (METI), private sector corporations, and other entities [7]. Since these ministries, except for MOFA, have limited ODA budgets for bilateral development cooperation for health [8], they are likely to use their public funds for bilateral development cooperation. Thus, in this study, the public funds, not the ODA funds contributed by MOFA, are referred to as “non-ODA” public funds.

In the framework of ODA, Japanese embassies overseas in collaboration with JICA’s country offices play a principal role in identifying the needs of the recipient country for new projects based on the request survey every year. ODA scheme projects, such as technical cooperation and grant aid, are formulated based on the official requests from the recipient country through diplomatic channels [9]. However, this framework does not apply to projects funded by non-ODA public budgets. Therefore, a Japanese embassy and the governments of the recipient country may not always keep a record of projects funded by Japanese non-ODA public budgets. Although the Headquarters for Healthcare Policy urges the ministries to coordinate with one another under the “the Basic Design for Peace and Health,” the overall picture of health cooperation implemented on the ground at a country level and funded by both ODA and non-ODA budget have not been clearly mapped or understood.

Therefore, there is a need to understand how Japan’s publicly funded cooperation for health by both ODA and non-ODA public funds address HSS in the context of the recipient country’s health system priorities. In this study, we took the case of Vietnam, the third-largest recipient of Japan’s gross bilateral ODA in 2018 [3], and where Japan is the largest donor [10]. Vietnam and Japan have had close economic and cultural relationships and exchanges over the last few decades. The first aim of this study is to map funders and project implementation for Japan’s publicly funded projects by both ODA and other non-ODA public funds in the area of development cooperation for health in the case of Vietnam. The second aim is to describe the intervention areas and focus of Japan’s publicly funded projects by using the WHO’s framework of health system building blocks. It further attempts to identify the collaborations between the various Japanese actors concerning their projects and project alignment with the overall strategy of *“the Basic Design for Peace and Health.”* Based on the results of this study, we discuss unique policy implications for country-specific cooperation strategies for health in Vietnam and beyond.

## Methods

### Scope of analysis and data collection

A cross-sectional analysis of the Japanese publicly funded projects (including both ODA and non-ODA expenses) related to cooperation for health in Vietnam was conducted. The analysis included health-related projects that were being implemented in Vietnam during the month of December 2016. Projects in the fields of long-term care and social security were also included in the scope of analysis as these areas are closely related to health. An internet search to identify the projects was conducted between July and September 2017; the details of this search are presented in an additional table file (see Additional file 1). Research projects identified on the target websites were included because the selected agencies of these websites mostly have both research and operational components. Survey projects that were identified in the target websites, aiming for formulating or promoting health cooperation, were included. The projects’ eligibility for the study was assessed by reviewing the project titles, basic information, and summary of projects. Projects that could not be confirmed to be publicly funded and projects that were a part of a larger research project were excluded.

**Fig. 1:**
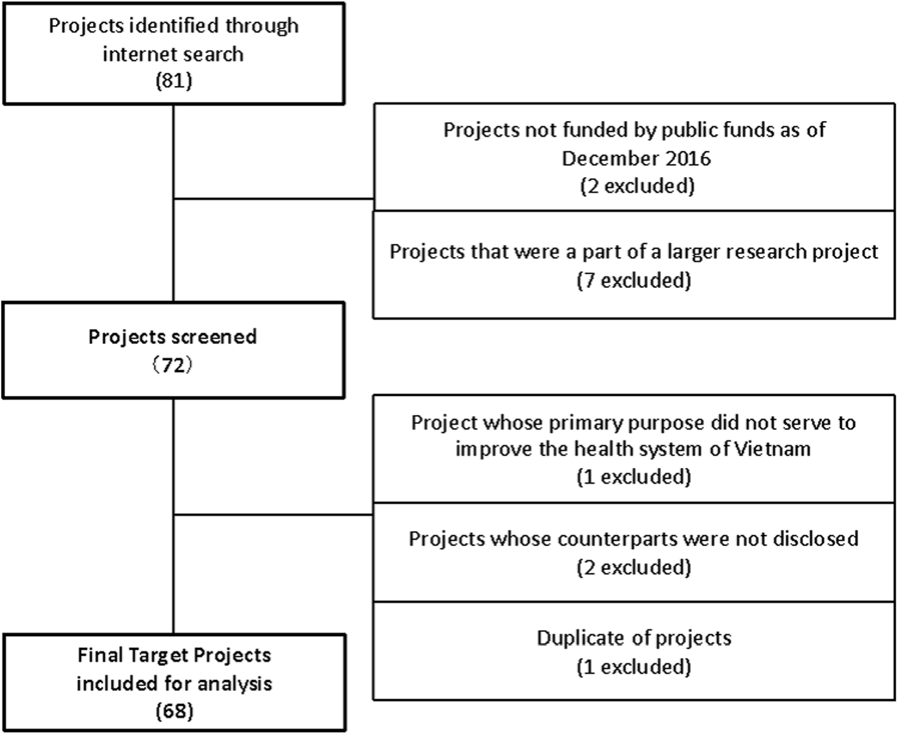
Selection process of the target projects

The remaining projects were further screened by assessing additional information on project publications. During this screening process, the projects whose primary purpose did not serve to improve the health system of Vietnam and projects whose counterparts had not been disclosed were excluded. The selection process of the target projects is summarized in Fig. 1. The basic information of the target projects including the project summaries (details in Additional file 1) was collected and assessed. Wherever needed, additional information was collected face-to-face, via phone, or via e-mail from the relevant scheme operating organizations responsible for the projects included in this study. The target projects were reviewed for any description of potential collaboration or harmonization promoted among Japanese funded projects using project publications.

### Framework of analysis

In this research, a framework based on the six building blocks of the World Health Organization’s (WHO) health system [11, 12] was adopted to conduct the analysis. As shown in Table 1, the six building blocks describe the core components of a health system, and have been adopted for assessments of global health initiatives and the health system at the country system level in previous researches [13–16]. In order to classify the projects identified in our study, the six building blocks were broken down into the three tiers of “building blocks,” “function,” and “activities component” based on past literature and expert consultation [17–20].

**Table 1:** Activities Categorized by WHO’s Six Building Blocks of Health Systems

Building Block	Function	Activity Component
**Health service delivery**	Service availability	Infrastructure (Facility (number and distribution))
		Health workforce availability
		Service utilization (inpatient and outpatient visits)
	General and specific service readiness	Infrastructure/ amenities
		Supply/ equipment
		Standard precautions
		Laboratory tests (diagnostics)
		Medicines and commodities
		Staff and training (service standardization, program specific)
		Diagnostic skills and training (capacity standardization, program specific)
	Service quality	Service improvement (coverage, effectiveness, safety, patient-centeredness, timeliness)
**Health workforce**	Training, recruitment, and retainment	Human resources (HR) development plan
		Training
		HR performance and management
		Education
		Regulations (licensing and accreditation)
		HR production, recruitment, and data management
**Health information systems**	Generation of population and facility-based data	Health survey
		Census
		Civil registration
		Disease management information system
		Health facility reporting
		Public health surveillance system
		Health system resource tracking
	capacity for analysis, synthesis, and validation of data	Public health threat response system
		Performance tracking
		Monitoring and research
		Country, regional, and global data analysis
**Medical products, vaccines, and technologies**	Access to essential medical products, vaccines, and technologies	Policy development (national policies, standards, guidelines, and regulations) for essential medicines, vaccines, and technology
		Procurement of essential medicines and vaccines
		Quality assessment of priority products
		Support for rational use of essential medicines, commodities, and equipment
		Technological assessments (stimulate development, testing and use of new products, tools, standards, and policy guidelines)
		Insurance coverage (% population covered, price and cost of medicine)
		Essential medicines (14 medicines)
**Health system financing**	Financing	Collection of revenues (domestic and international funding sources)
		Risk pooling (benefit coverage and entitlement)
		Purchasing of services
**Leadership and Governance**	Policy, Leadership, Governance	National policy and strategies, action plans (including disease/program specific ones)
		Accountability
		Working with external partners

We deferred to definitions from the WHO reference materials to determine the categorization of activities that can span across two building blocks [12, 18]. For example, we identified pre-service and in-service training as a part of the “health workforce” building block if the activity resulted in newly qualified health professionals or cadres, and as a part of the “health service delivery” building block if the activity was identified as quality improvement and service enhancing training for health professionals. The projects were further “recategorized” as a separate variable to account for multiple building blocks a project may be covering. For instance, projects labeled “health service delivery” as their primary categorization were labeled with an additional categorization of “health workforce” if the projects addressed this component as well.

We also described whether the projects either reported output or outcome indicators in order to understand the types of measurement used for project evaluation. Outcome indicators are “changes brought about to target populations or beneficiaries by means of intervention output by projects,” and output indicators are “outputs, capital goods, and services generated as a result of assistance or changes brought about by assistance” [21–23].

### Project classification and data analysis

The targets, outcomes, and indicators of each project were carefully reviewed to classify the projects based on the analysis framework of the six building blocks adopted for this study. Each project’s counterpart organizations in Vietnam were identified based on the documents reviewed. These organizations were classified as one of the following types of institutions: governmental administrative bodies, public medical facilities, research institutions, educational institutions, and others such as non-governmental organizations and private hospitals. In Vietnam, there are four levels of health administration for healthcare services and management: central, provincial, district, and commune [24]. In this study, the level of health administration for the counterpart organizations of governmental administrative bodies and public medical facilities was classified into the three levels of central, provincial, and local. Tertiary level hospitals, categorized as Level 1 hospitals in Vietnamese health administration, were classified as the central level of health administration in this study [25]. Centrally-controlled cities such as Hanoi city were classified at the provincial level. The local level of health administration included city, district, and commune levels. The commune people’s committees were categorized under the local level because of the role of such committees in the official management of the health system. If a project did not fit into any of these categories, it was classified as “others.”

Coding was implemented by two researchers independently (SL, MU). Each project was analyzed based on the framework adopted for this research. When the two researchers had different opinions on the classification, a discussion was held to reach a consensus, whose outcome would then be adopted. When the classification criteria were unclear, the research members (SL, MU, AI) discussed further to segment and review the criteria. Some projects showed indications of being implemented at several health administration levels simultaneously. In such cases, the number of projects and financial contribution by level were weighted proportionately to the number of levels (multiplying 1/*n* to the financial contribution when there are *n* levels and redistributing equally to each level). Data on the six building blocks identified the main block, which was directly used to identify the “main six building blocks.” Meanwhile, as some projects were categorized under several building blocks, the same adjustment procedure was taken to proportionately weight the number of building blocks, in order to correspond to the complex reality of health projects.

First, the annual budget for each project was estimated using the total project cost per year and converted to USD (1 $ = 108.79 ¥) based on the Organisation for Economic Co-operation and Development (OECD) exchange rate as of 2016. Since the project budgets of some projects were not disclosed, the estimates for those projects were calculated based on the maximum applicable amount of the project budget described in the scheme-related documents such as project application information. A frequency distribution of the number of projects was created, and financial contributions by each of the main six building blocks as well as their proportion out of the total contribution of projects assessed in this study were estimated. Second, the aggregated financial contributions and their proportion out of the total were described by the building blocks and the level of health administration. Last, visualization flow charts were created to track resource allocation by scheme operating organization, health administration level, main six building blocks, and recategorized six building blocks.

Preliminary results were shared with the relevant ministries and agencies and ODA implementation organizations to assess data accuracy and improve the data contents. In each case, the comments and information obtained were recorded in research notebooks and the project classification was revised as needed based on the feedback received.

## Results

A total of 81 projects were retrieved through the internet search, and 68 projects were deemed to be eligible for inclusion in the final analysis. These 68 projects under 15 types of schemes were managed by 7 different scheme-operating organizations and funded by 5 ministries (see Table 2). Of these, 44 (64.7%) were ODA projects funded by the Ministry of Foreign Affairs, whose schemes were operated by the Japan International Cooperation Agency (JICA) (37 projects), Embassy of Japan in Vietnam (six projects), and jointly by JICA and Japan Agency for Medical Research and Development (AMED) (one project). The remaining 24 projects (35.3%) were non-ODA projects funded by the MHLW; METI; Cabinet office; and the Ministry of Education, Culture, Sports, Science and Technology. These projects’ schemes were operated by the MHLW (11 projects), National Center for Global Health and Medicine (six projects), AMED (four projects), METI (two projects), and Japan External Trade Organization (one project).

**Table 2:** Project funding agencies and their schemes, 2016

Funding Ministry	Scheme Operating Organization	Identified Projects		Schemes		ODA
		(n)	(%)	Scheme name	(n)	
MOFA	JICA	37	54.4	Loan Aid	5	○
				Technical Cooperation Projects	4	○
				JICA Partnership Projects	6	○
				Japan Overseas Cooperation Volunteers	17	○
				Public-Private Partnership	5	○
	JICA/AMED	1	1.5	Science and Technology Research Partnership for Sustainable Development (SATREPS)	1	○
	Embassy of Japan in Vietnam	6	8.8	Grant Assistance for Grassroots Human Security Projects	6	○
MHLW	MHLW	11	16.2	The International Promotion of Japan’s Healthcare Technologies and Services	11	
	NCGM	6	8.8	Operational Funds	6	
METI	METI	2	2.9	Program to Promote Medical Technologies and Services (*Iryo Gijyutsu Sabisu Kyotenka Sokushin Jigyou*)	1	
				Survey Programs for Promotion of High-Quality Infrastructure System Development in Overseas (*Shitsu no Takai Infura Shisutemu Kaigai Tenkai Sokushin Chosa-tou Jigyo*)	1	
	JETRO	1	1.5	Survey Projects (*Chosa Jigyou*)	1	
Cabinet office /METI/MEXT /MHLW	AMED	4	5.9	Japan Initiative for Global Research Network on Infectious Diseases (J-GRID)	1	
				Research Program on the Challenges of Global Health Issues	2	
				Research Program on Emerging and Re-emerging Infectious Diseases	1	
**Total**		**68**			**68**	

The Japanese organizations and project actors that implemented these projects in the field were central and local governments, the ODA implementation agency, medical institutions, healthcare professional associations, research institutions, higher educational institutions, private sector companies, social welfare service institutions, non-profit organizations, and independent administrative agencies. Their major counterparts in Vietnam were the governmental health administration and public medical facilities. Of the 68 projects, we were able to identify the entire project implementation period for 62; the mean was 24.9 months, ranging from 5 months to 5 years. Among the activity components of each project that were used as the basis for categorization into the six building blocks, the largest number of projects addressed service improvement (19.7%) and followed by training of health personnel (17.6%). The details of the qualitative information obtained from the projects are presented in an additional file (see Additional files 2 and 3).

Based on the available project reports, there was limited description regarding the collaboration between Japan’s different publicly funded projects. For example, some JICA’s PPP survey reports described potential collaboration with ODA projects in the future.

**Fig. 2:**
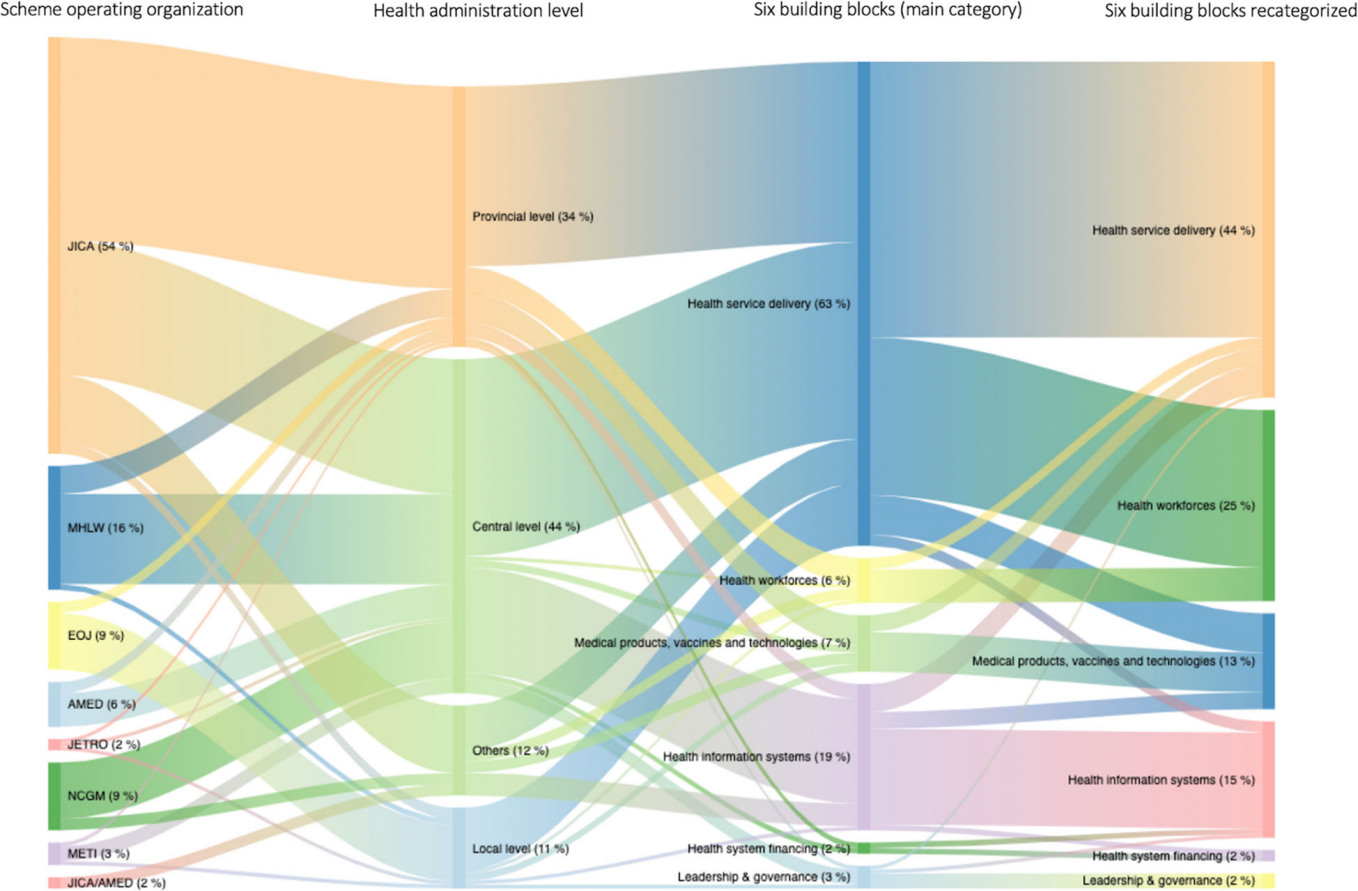
Flow of proportions of the number of projects implemented by Japanese public funds to Vietnam, by source, health administration level, and purposes categorized into the World Health Organization’s six building blocks (the main category and recategorized building blocks). *JICA* Japan International Cooperation Agency; *MHLW* Ministry of Health, Labour and Welfare; *EOJ* Embassy of Japan in Vietnam; *AMED* Japan Agency for Medical Research and Development; *JETRO* Japan External Trade Organization; *NCGM* the National Center for Global Health and Medicine; *METI* Ministry of Economy, Trade, and Industry. Note: the proportions were estimated by using a total number of projects (68 projects) that were identified from publicly available resources as the denominator

Figure 2 shows the flow in proportion to the number of projects by scheme operating organizations, health administration level, and purposes categorized into the main and recategorized building blocks. Most projects were operated either at the central level (44%) or at the provincial level (34%). About 60% of the projects implemented at the central level were non-ODA projects, while the rest were ODA projects. ODA projects constituted 71% of the total projects implemented at the provincial level, while the rest were non-ODA projects. ODA projects constituted 80% of the total projects implemented at the local level, with the rest being non-ODA projects. Among the main six building blocks, the largest number of projects was concentrated in the area of health service delivery (63%), followed by health information systems (19%). Among the recategorized six building blocks, the focus area of the largest number of projects was health service delivery (44%). This was followed by the focus areas of health workforces (25%); health information systems (15%); medical products, vaccines, and technologies (13%); health system financing (2%); and leadership and governance (2%).

Almost half of the projects focusing on health service delivery as the main building block identified central hospitals as their counterparts. Among these, 15 projects, in particular, were carried out with two specific central hospitals. The projects for this building block focused on infrastructure development and the technical improvement of medical services. For example, some projects operated by JICA and MHLW strove to improve inter-professional work relating to the quality of medical services and patient safety in order to enhance overall hospital management. Other projects in this building block category sought to enhance medical services relating to rehabilitation, stroke, and diabetes mellitus. In particular, under the JICA’s Japan Overseas Cooperation Volunteers scheme, 17 projects dispatched healthcare personnel such as occupational therapists and physical therapists, and other personnel providing care and support for disabled children to health facilities, mainly at the provincial level. Two-third of the projects for health service delivery incorporated health workforce activity in order to improve the quality of health services. This trend has led to an increase in the proportion of projects focusing on the health workforce in the recategorized building block.

With regard to the building block of health information systems, a majority of projects that were non-ODA projects were collaborative health research between Japanese and Vietnamese research institutions. The AMED projects advanced research on various infectious diseases, which contributed to the improvement of knowledge on the diagnosis, prevention, and treatment of infectious diseases between Japanese universities and Vietnam’s national health institutes working on infectious diseases. The National Center for Global Health and Medicine implemented mostly clinical research projects with the central hospitals. Under the METI’s scheme, the Japanese private sector collaborated with a Vietnamese central hospital to launch a project operating medical services and radiological examinations efficiently by utilizing Japanese healthcare information and communication technology.

Several projects contributed toward medical products, vaccines, and technologies by providing medical equipment for health facilities and promoting Japanese health technologies. An example of this includes JICA’s project that aimed to enhance the local production capacity of a combined measles and rubella vaccine in Vietnam.

### Project budget

According to information disclosed by 39 projects on their project budget, a total of 9.3 billion ¥ (US$ 85.4 million) was invested for health cooperation in Vietnam in 2016 (Table 3). The median project budget was 10.0 million ¥ (US$ 91,918) in 2016, ranging from 4.0 million ¥ (US$ 36,967) to 5.7 billion ¥ (US$ 52.6 million).

**Table 3:** Annual budget proportion by six building blocks, 2016

Six Building Blocks	Total Projects		Projects with budget information		
	n	%	n	USD (mil)	%
Health Service Delivery	43	63.2	26	62.3	73
Health Workforce	4	5.9	4	1.4	1.7
Health Information System	13	19.1	2	0.9	1.1
Medical Products, Vaccines and Technologies	5	7.4	4	16.4	19.2
Health System Financing	1	1.5	1	3.5	4.1
Leadership and Governance	2	2.9	2	0.8	1
**Total**	**68**	**100**	**39**	**85.4**	**100**

1 $ = 108.79 ¥ (Organisation for Economic Co-operation and Development exchange rate, 2016)

**Fig. 3:**
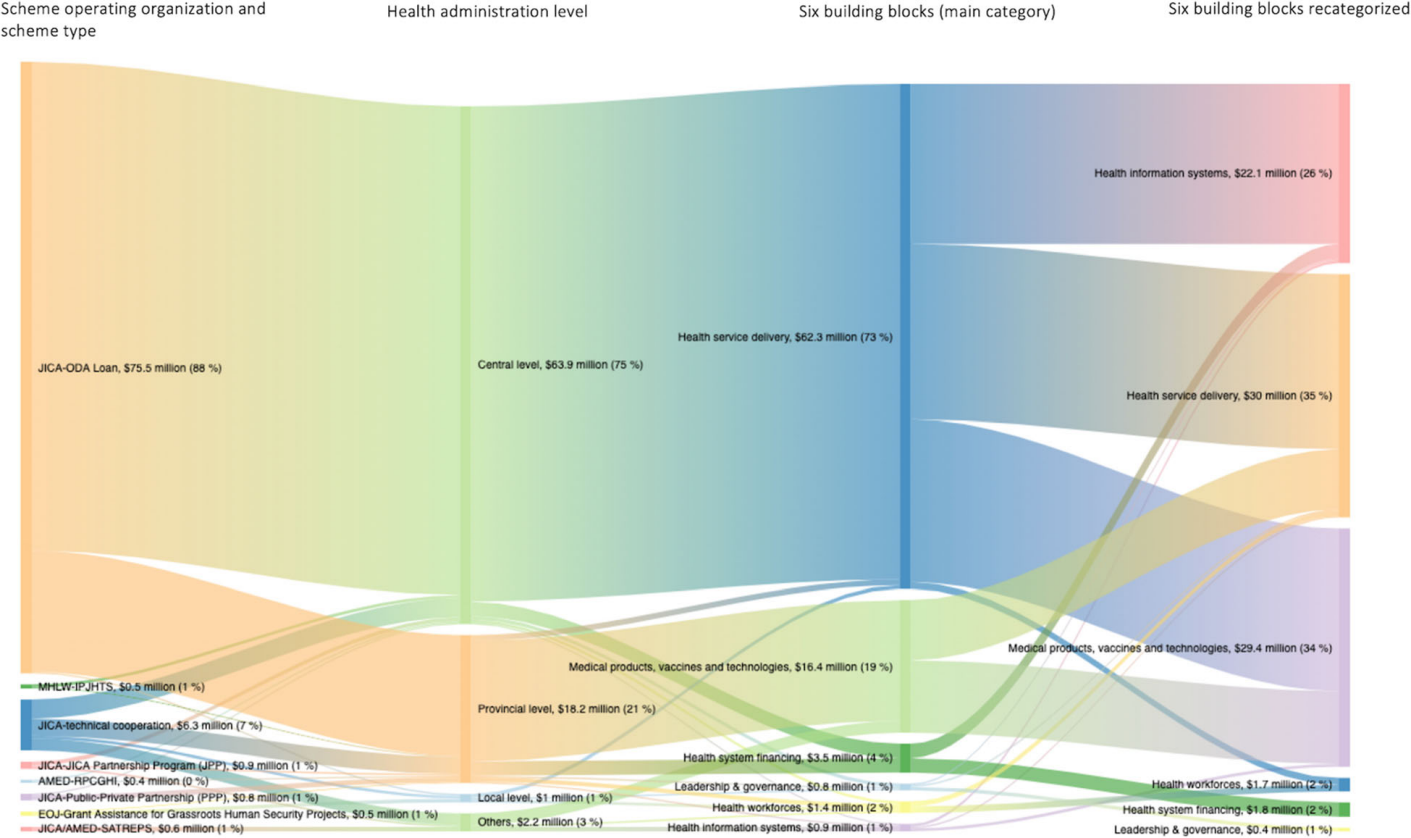
Flow of annual project budgets from both Japan’s ODA and non-ODA public funds that were implemented for health projects in Vietnam in 2016, by scheme operating organizations and their scheme types, health administration level, and purposes categorized into WHO’s six building blocks (the main category and recategorized building blocks). (2016 USD). *JICA* Japan International Cooperation Agency; *MHLW* Ministry of Health, Labour, and Welfare; *IPJHTS* The International Promotion of Japan’s Healthcare Technologies and Services; *PPP* Public-Private Partnership; *EOJ* Embassy of Japan in Vietnam; *AMED* Japan Agency for Medical Research and Development; *SATREPS* Science and Technology Research Partnership for Sustainable Development. Note: IPJHTS is an unofficial abbreviation created only for the purpose of this paper. The proportions were estimated with a total budget of resource allocation that were identified from publicly available resources (39 projects) as the denominator

Figure 3 shows the flow of the annual project budgets, by scheme operating organizations and their scheme types, health administration level, and purposes categorized into the main building blocks and recategorized building blocks. By scheme type, the largest project budget was JICA’s loan aid scheme (88%) followed by JICA’s technical cooperation scheme (7%). The majority of the project budget was distributed to the central level of Vietnam’s health system (75%), followed by the provincial level (21%) and the local level (1%). This is mainly due to the largest investment being utilized in the construction of a new central hospital by JICA’s loan aid; this project aimed to alleviate overcrowding of patients in currently the largest central hospital in a certain region of Vietnam. On assessing project budget based on the six building blocks, most of the budget was found to be concentrated in the area of health service delivery (73%), followed by medical products, vaccines, and technologies (19%). When recategorized, the budget mainly dispersed among health service delivery (35%), medical products, vaccines, and technologies (34%), and health information systems (26%). The proportion of annual project budget categorized by the WHO’s six building blocks and level of health administration is shown in Additional file 4.

### Project outcome indicators

**Fig. 4:**
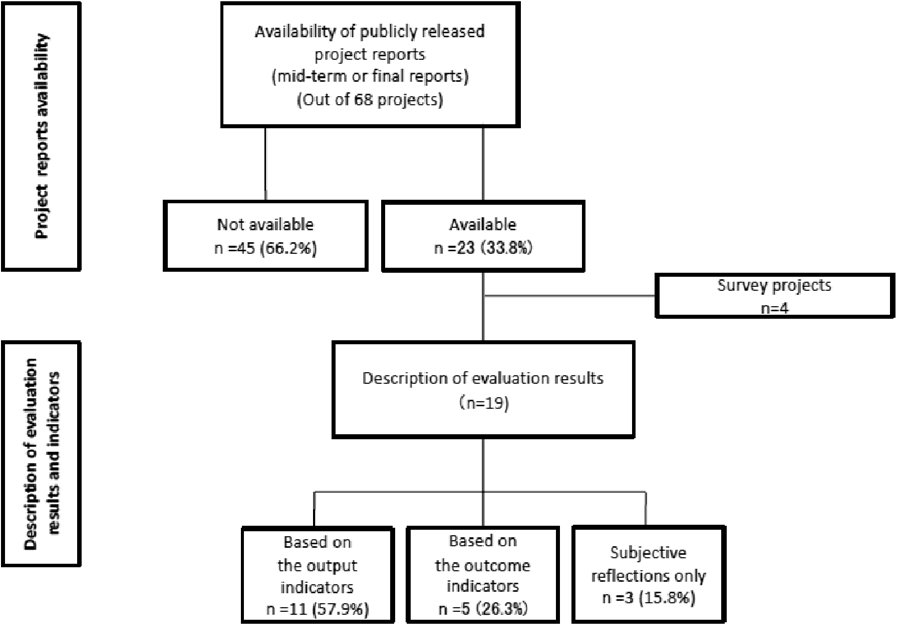
Assessment of reporting project evaluation with the project outcome indicators

Figure 4 illustrates the assessment of the description of evaluation results and indicators for the projects. Among the 45 projects that did not have a publicly available final report in September 2017, 32 projects were still under implementation, while the remaining 13 projects had been completed within a six-month period prior to September 2017. Mid-term or final reports were publicly available for only 23 projects, out of which four were survey projects and were excluded from further assessment. This is because these surveys were conducted for assessing the situation and needs relating to the target topic for formulation of future projects; thus, their project outcome did not satisfy our indicator “changes brought about to target populations or beneficiaries.” Out of the 19 projects with confirmed descriptions of evaluation results, 11 projects (57.9%) published evaluation reports with the results based on output indicators, and five projects (26.3%) published reports with the results based on objectively measurable outcome indicators. Among the five projects, three were JICA’s projects. Their results included technical transfer of measles-rubella combined vaccine (MR vaccine) production in Vietnam by confirming that Vietnamese staff acquired sufficient levels of techniques for each process of the vaccine production and quality control. In another JICA project, progress of the referral system was observed by confirming the issue of related Vietnamese circular, referral performance, local healthcare activities among healthcare facilities to strengthen their healthcare service capacity, and the number of assigned local personnel for the related roles. In a JICA-AMED project, the evaluation confirmed the spread mechanism of multidrug resistant bacteria based on scientific evidence, as well as the progress of developing the monitoring system in the country. Remaining projects were NCGM’s research and operational projects on noncommunicable diseases (NCDs) that reported results such as the prevalence of overweight or obesity in school children through lifestyle interventions. The remaining three project reports (15.8%) described subjective impressions and reflections without an outcome- or output-based objective indicator.

## Discussion

### Overall picture of Japan’s development cooperation for health in Vietnam

To our knowledge, this is the first study that identified an overall picture of Japanese development cooperation for health in Vietnam for both ODA and non-ODA publicly funded projects. This is also the first study to assess Japanese health cooperation using the WHO’s framework of six building blocks. A total of 68 projects were funded by MOFA and four other ministries; and implemented by a wide range of entities including governmental agencies, the ODA implementation organizations, medical institutions, academia, for-profit businesses, and civil society organizations. These entities mobilized their technical expertise, with a heavy focus on health service delivery, in cooperation with Vietnamese counterparts from central, provincial, and local levels. This involvement of diverse resources supported the aim of Japanese policies that have endeavored to utilize Japan’s non-ODA public financial resources and various other resources in development cooperation [4]. Improving healthcare services was the major activity that the target projects collaborated on with their Vietnamese counterparts including health facilities, health administration, and social welfare services for disabled people and children. The range in scope of the Japanese projects are considered to be in line with the Vietnamese health policy aimed at improving the quality of medical services and rehabilitation [26].

The projects in our study sample addressed a wide range of health issues across all six building blocks of the Vietnamese health system. In the categorical analysis of the main six building blocks, health service delivery accounted for the focus of more than 60% of the projects, followed by health information systems. This trend is similar to a previous study that reported service delivery and health information systems to be the most common interventions in five African countries [27]. In contrast, the recategorized building blocks showed a clear change in terms of the increased proportions of the other building blocks. This reveals that the target of Japanese funded projects was not necessarily concentrated in the area of health service delivery block. Through the recategorization, it became clear that one-quarter of the projects were devoted to the health workforce. Similar to our research results, health service delivery and health workforce were included among the three major approaches to strengthen the health system of eight countries including Vietnam in a study analyzing Germany’s bilateral cooperation with these eight countries [28]; however, the funding resources of this study included only ODA. Unlike Germany, whose most prioritized focus area was leadership and governance, only three ODA projects in our study focused on leadership and governance as well as health financing. These blocks play a significant function in advancing UHC [29]. Leadership and responsible stewardship are essential in directing an efficient health system at the national level [30]. Japan’s cooperation in these areas is considered significant irrespective of the number of projects implemented in Vietnam. This is because they align with the country’s needs in accordance with Vietnamese policies on efficient health financing towards UHC and improvement of medical services in mountainous and rural areas for equitable healthcare access [26].

### The role of Japan’s assistance and health cooperation for Vietnam

Japan’s health cooperation for Vietnam observed in this study aligns with the following broader role of Japan’s aid policy for Vietnam, a country observing steady economic growth over the past decades. Japan’s aid aims to support Vietnam in achieving sustainable development by strengthening their international competitiveness, overcoming vulnerability, and creating a fair society towards industrialization [31]. Health cooperation is one area to support Vietnam in improving the social aspects and living standards in response to the negative consequences brought on by the economic development [31]. Therefore, strengthening the health system for UHC according to Vietnam’s needs and mutual interests would be beneficial in realizing equitable and sustained improvements across health services and health outcomes [11]. From these perspectives, one successful model of health cooperation would be the JICA’s technical cooperation project for the measles-rubella combined vaccine production that was implemented with the cooperation of the Japanese private sector and transferred Japan’s expertise and technology. This helped Vietnam acquire skills in domestic vaccine production that meets international standards. As a result, the local capacity to produce vaccines helped boost vaccination among children and pregnant women; thus, improving the health of children nationwide by protecting against vaccine-preventable infectious diseases. Based on Japan’s policies, we elaborate on the characteristics of their development cooperation for health in Vietnam as well as whether their publicly funded projects fit in the general Vietnamese health policies.

#### Strong cooperation with the central hospitals

This study observed health cooperation with the central hospitals to be a major characteristic of Japan’s development cooperation for health in Vietnam. Almost half of the projects involved Vietnam’s central hospitals; specifically, two central hospitals in the large cities were involved in 25 projects. The budget distribution was also the largest at the central level. However, this is due to JICA’s loan aid, which is different from the grant aid, extending financial assistance to the recipient countries without repayment. The loan aid scheme facilitates the efficient use of borrowed funds and the proper supervision of the projects that the recipient countries finance, thereby supporting the ownership of the recipient countries in the development process [32].. Among the target projects, our study observed no project under the grant aid scheme at the central and provincial levels of health administration in Vietnam. Therefore, the application of the loan aid seems relevant for Vietnam, which is a lower-middle-income country and has achieved significant economic growth in past decades [33].

The concentration of projects in central hospitals can be considered a result of the historical background of Japan’s ODA with Vietnam. After resuming Japan’s ODA in 1992, JICA implemented various projects targeting central hospitals in the northern, central, and southern regions of Vietnam. Several projects were conducted by both the loan aid scheme for infrastructure development of hospitals and the technical cooperation scheme for improving the quality of medical services and hospital management and developing human resources [34]. Since these central hospitals play a major role as regional training hubs, implementing health cooperation projects with these hospitals is expected to develop human resources for health. Further, it will have a spillover effect where there is a transfer of knowledge and skills to hospitals at local levels under Vietnam’s health policy [25, 35]. Given these benefits, it is considered worthy to contribute to hospital infrastructure through a loan-aid scheme under the Japanese ODA funds in conjunction with technical assistance schemes.

It is likely that the long-term partnership of Japan with these central hospitals through ODA resulted in their being considered as co-implementing institutions when starting a new project. Accordingly, various projects have been launched with these central hospitals by utilizing ODA schemes such as the public-private partnership (PPP) scheme of JICA as well as the relatively new non-ODA schemes of MHLW and METI for promoting Japan’s medical skills and technology internationally. Clinical research projects have also been launched as a new area of collaboration between Japanese institutes and Vietnamese central hospitals using MHLW funds. Targeting the central hospitals in these clinical research areas may involve factors with relatively abundant human resources who can manage clinical research and equipment.

This study observed that almost half of the projects worked to improve service delivery through cooperation with the central hospitals. In addition, the majority of Japanese resources implemented at the central level were non-ODA projects, especially projects funded by MHLW. In 2014, the Japanese MHLW and the Vietnamese Ministry of Health agreed and signed the Memorandum of Cooperation in the field of healthcare [36] with the aim to strengthen cooperation based on mutual interest, such as human resource development for health professionals as well as the introduction of advanced medical technology. These areas could mobilize Japanese expertise and experiences in line with the Basic Design for Peace and Health policy.

The close cooperation with these central hospitals can be utilized by the Japanese funded projects to proceed to the next stage of cooperation, aimed at addressing the major challenges in health service delivery in Vietnam. A plan of Vietnamese Ministry of Health for people’s health protection, care, and promotion between 2016 and 2020 aimed to reduce the overcrowding of patients, particularly at the central hospitals, which has been a long-standing challenge in Vietnam [26, 37]. Several measures were proposed in this five-year plan, such as increasing the number of health facilities at all levels, developing a satellite hospital network, and enhancing technical transfer between medical institutions across health administration levels by rotating human resources for health. However, this study found that only a few Japanese funded projects worked to improve medical services at provincial or local levels by linking the central and provincial level health systems. For example, an MHLW project collaborated with a Vietnamese central hospital and a medical educational institute for implementation of Vietnam’s policy on the ground by strengthening a rotation training system for newly graduated physicians working at provincial hospitals. Additionally, a JICA project that aligned with Vietnam’s health policy, called the “Direction Office for Healthcare Activities (DOHA),” worked to strengthen the referral system between medical facilities at different health administration levels in mountainous areas, and promoted clinical skill guidance and supervision activities among these medical facilities [25].

Since the Japanese funded projects majorly concentrate on the central hospitals, a greater number of projects funded by Japan should leverage the strengths of this cooperation with the central hospitals. Doing so will allow the central hospitals to, efficiently and simultaneously, transfer advanced and cutting-edge technical skills to the provincial level health facilities. For example, Vietnam’s Satellite Hospital Project prioritized several specialties such as oncology, traumatology, and cardiology, and has actively promoted the transfer of its advanced medical and surgical skills from the central to provincial hospitals [25]. Carrying out projects that align with core Vietnamese priorities and policies jointly with the central hospitals could further enhance Vietnam’s sense of ownership and contribute toward a sustainable health system. These efforts would allow more patients to receive quality medical and healthcare services locally, which, in turn, would reduce the workload of the central hospitals.

#### Addressing health disparity by improving primary health care through further cooperation

In order to reduce health disparities between the urban and rural populations, effective provision of appropriate healthcare services at the community level in the rural areas of Vietnam is a key challenge [26, 38]. Major efforts have been made by the Vietnamese Ministry of Health’s initiatives to improve Primary Health Care (PHC) such that healthcare services are accessible to all people who need it [39, 40]. Quality and accessible primary healthcare is essential for achieving UHC [41, 42]. However, this study’s analysis revealed that only 10% of the projects were conducted at the local level, and these projects were mainly ODA projects. For example, the Embassy of Japan in Vietnam allocated their Grant Assistance for Grassroots Human Security Projects for the expansion of five commune health centers in the rural areas of Vietnam. A survey project was also initiated for a need assessment in underserved local communities of rapid diagnosis test kits for the hepatitis B virus invented by a Japanese company under the JICA’s PPP scheme. Newborn babies and their mothers were identified as the prioritized groups and beneficiaries nationwide. Such rapid, affordable, and easy diagnosis tools can have significant positive impacts on an effort to secure the health of children and mothers, especially in the remote and isolated communities.

Moreover, various innovative approaches should be proactively initiated so that those providing clinical technical support to health personnel in Vietnamese health facilities can benefit from the improvement of medical services at the local level. Through PPP, several projects at the central hospitals promoted both technical skills transfer of medical services and utilization of Japanese medical devices for efficient medical services. For example, a clinical tele-consultation system between a group of physicians from Vietnam and Japan was developed to improve child cancer diagnostic skills in Vietnam. This kind of telemedicine could be applied to the development of remote clinical consultation systems for rural or hard-to-reach areas in Vietnam. Since grassroots-level clinical counseling and technical support is one of the training and teaching tasks of the central and upper-level hospitals in Vietnam, this characteristic of domestic technical transfer could be promoted efficiently with the utilization of information and communication technology. Currently, the Vietnamese government sees the importance of remote consultation because it has become helpful during the coronavirus disease 2019 (COVID-19) pandemic. Remote consultation has helped health facilities at the grassroots receive timely technical support for the diagnosis and treatment of COVID-19 patients from higher-level hospitals [43].

At the same time, community-based health promotion as well as elderly care and support are also imperative to respond to the increasing prevalence of NCDs and the needs of an aging society [39]. Based on the qualitative information of the projects, 31 projects (45.6%) of the original 68 addressed NCDs. Among those, 19 projects were implemented with counterparts at the provincial or local levels. Such efforts on strengthening health services, including the prevention of NCDs, through collaboration between domestic and international partners are expected to continue. Vietnam is one of the most rapidly aging countries in Asia [44, 45]. Thus, the role of Vietnam’s local communities in healthcare is critical as they can take on responsibility for providing comprehensive and easily accessible care and support to the elderly in their communities [46, 47]. In July 2019, the Japanese and Vietnamese governments signed a Memorandum of Cooperation in the field of healthcare [48]. This memorandum emphasized the promotion of a Japanese policy called the Asia Health and Wellbeing Initiative that aims to foster development of long-term care for elderly people through the PPP approach and human resource exchange programs. The Asia Health and Wellbeing Initiative, which is led by the Japanese government, should be taken as an opportunity to boost Japan’s development cooperation for community based long-term care and support for the elderly in Vietnam [49].

This study identified some of the specificities of Japanese cooperation with an emphasis on tertiary healthcare advancement as well as technological and scientific innovations in Vietnam. It is likely that significant hospital investments are an appropriate funding target for the donors, depending on the broader context of the Vietnamese health system. The same applies to the joint research projects with an aim to enhance evidence-based clinical interventions. This may possibly suggest that Japanese funded projects in Vietnam are prioritizing technological utilization and advancement of tertiary care facilities over allocating resources to PHC at the local level. This raises the following questions: As a result of this prioritization, could the Japanese development cooperation be interrupting, or indirectly pre-determining the way Vietnam’s domestic health funds are being allocated to PHC? Do the specificities of Japanese cooperation justify the donor country’s focus on advanced technical skills and technological utilization as long as the Vietnamese government allocates sufficient funds to PHC? To answer these questions, we would need to conduct a complete and thorough review of the health sector progress and performance in Vietnam. Joint health reviews among the Ministries of Health of recipient countries and their health sector partners would serve as extremely informative opportunities for Japan as a donor country; it would allow Japan to reflect upon the recipient country-specific health cooperation strategies.

### Ensuring efficient and effective overall development cooperation for health

#### Monitoring and evaluation across schemes

Despite the fact that Japan devotes a large portion of its public funds to bolster the health scenario in Vietnam, 26.3% of the projects that we were able to confirm the descriptions of evaluation results, published reports with the results based on objectively measurable outcome indicators. The evaluation results based on outcome indicators were more likely to be reported if the project period was longer than 4 years. However, evaluation results based on only the output indicators were reported by projects that culminated within 3 years and where a majority of them were implemented for only 1 year. A sufficient project period, therefore, is needed to allow for measurement and evaluation, which should focus on the outcomes of cooperation that primarily aims to improve the health of the people [50].

Our study observed some JICA technical cooperation projects that reported progress or results with objectively measurable outcome indicators. This is probably because JICA conducts an evaluation of major schemes, loan-aid, grant-aid, and technical cooperation in accordance with the Development Assistance Committee (DAC) evaluation criteria established by the OECD as well as JICA’s own rating system [51]. JICA’s evaluation guidelines describe the schemes and projects targeted, and the conditions for the projects that require external evaluation. However, this evaluation framework that is based on DAC criteria may not necessarily be utilized for ODA projects with relatively small budget sizes [52]. We observed no publicly available midterm or final project reports for some JICA projects that were from non-major schemes with relatively small budget sizes even after project completion. It is possible that some project reports are not open to the public or were still being prepared at the time of our study. Regarding non-ODA funding projects, we did not find any evaluation framework. However, a relatively new scheme of MHLW was developing their evaluation framework at the time of our data collection period [53]. The presence or absence of an evaluation framework seems to depend on the scheme and funding ministries [21], but the accountability for the use of public funds regardless of resources should be considered. Under these circumstances, it is desirable to establish an independent body of external experts for technical guidance on monitoring and evaluation across schemes.

In this study, numerous projects by the Japanese resources were identified, which were implemented simultaneously to improve the health sector of Vietnam. Japanese and Vietnamese actors cooperate in each project at multiple levels of the Vietnamese health system to meet Vietnam’s needs and mutual interests. However, this study was unable to capture the synergetic effects produced by potential collaboration or harmonization between these projects. It implies that there are some gaps between policy and implementation at the field level. In addition, there were methodological limitations in trying to capture the synergic effects of collaboration or harmonization in this study. Although we attempted to assess these aspects based on the project’s qualitative information, due to the cross-sectional nature of this study, we were unable to complete the process because the final reports of most projects were not publicly available. In order to assess the collaboration efforts across the Japanese funded projects, the final project report of all target projects should be reviewed. For projects that were completed, the final project reports should be published and made publicly available, which would allow all the stakeholders to evaluate their cooperation effectiveness. In addition, qualitative assessments including in-depth interviews of the project stakeholders would be extremely informative. A report review and evaluation would also create the necessary accountabilities for those who play integral roles in project planning, operational management, and supervision for the ongoing and future publicly funded *cooperation*. Furthermore, active partnerships with greater mutual goals between the project schemes across the ministries should be encouraged and maintained transparently if such effort has not yet been made by the Japanese scheme-operating agencies and institutions.

#### Domestic and international coordination mechanisms

Japan’s contributions were concentrated at the central level in the country that is facing tremendous challenges for improving PHC. While the impact on Vietnam, who has a relatively low dependence on external funding (approximately 3.0%) [54], may be minimal, the impact on aid-dependent countries could be large in magnitude. Therefore, the importance of coordination across domestic actors of the donor countries can be applied as an important lesson for the donors, including Japan, that work in countries with high external funding. In particular, Japan has begun to work on assistance to other countries not only with ODA, but also with non-ODA public funding. Therefore, coordination among the ministries’ schemes beyond those in charge of ODA is crucial. Such a mechanism may pose a challenge for the donor countries, but a model approach from the example of the health cooperation in Vietnam should be shown.

Prior to this study, aside from the data on ODA, there was no comprehensive data on Japan’s overall health cooperation projects for a recipient country. In this regard, it would be ideal for the Japanese government to set a country-specific mechanism for strategic coordination across the ministries for development cooperation for health. Such a step will not only aid the efficiency of Japan but also promote coordination among other donors and partners in Vietnam [55, 56] or any other country. Joint health sector reviews among the government, partners, and stakeholders from both domestic and international resources would be helpful for effective development cooperation for health. Mapping of development partners and international organizations was regarded as an essential activity under this joint work in Vietnam; it helps to show these partners and organizations whether and how their support is harmonized with other partners [57]. At the same time, representatives of donor countries and development partners are encouraged to understand other stakeholders from their country who work with their Vietnamese partners in the context of various cooperation based on diverse partnerships [58]. In fact, taking the current study as an opportunity, the Embassy of Japan in Vietnam has begun to release a list of Japanese health cooperation projects in Vietnam [59], by utilizing the project information gathered in this study.

#### Strengths and limitations of this study

Continuous improvement of objective assessment with internationally common frameworks is required for development cooperation for health. In particular, Japan’s cooperation approach is diversifying; therefore, it would be helpful to examine whether the cooperation is relevant to the recipient country’s health policy and efforts. There are only a very limited number of studies on Japan’s development assistance for health, and these studies have assessed ODA funding at a global level [8]. However, the current study is the first to reveal Japan’s unique approach for development cooperation for health at the recipient country level by mobilizing both Japan’s ODA and non-ODA public budget. In addition, this study provided qualitative information about the projects.

The six building blocks framework that was utilized in our study refers to the essential functions of health systems. Inter-dependence between blocks is the nature of a well-functioning health system [11, 13]. In addition, in the WHO framework of monitoring and evaluation of HSS, health service delivery components are listed on both outputs and outcomes of HSS and are the ultimate common pathway towards outcomes [60]. It says that health service delivery under outcomes consists of two aspects; one is the strict aspect of healthcare service delivery, and the other is health service delivery in a broad sense related to the reduction of risk factors or risk behaviors. In our research, the analytical framework was based on the health service delivery in the strict sense of the former. This may imply that defining the characteristics of each project with only a single block is unrealistic. Since utilizing this framework simply in analysis has a limitation [27, 28], interaction with other blocks should be considered in understanding the extent to which projects address each of the building blocks.

We countered this issue in our study through additional analysis using the recategorized six building blocks, which reflected a more comprehensive understanding of the focus areas of each project. This approach also had a limitation in that we equally redistributed the health administration levels and building blocks as we did not have the relevant information to determine the proportionate variation in the characteristics of these projects according to health administration level and building blocks. Elaboration of how the projects addressed each building block through their multiple approaches would require in-depth interviews with each project-implementing organization. Furthermore, we found that the categorization of projects by building blocks has limitations and may potentially lead to a biased understanding of what each Japanese funded project actually collaborated on. As we provided the additional qualitative information based on the publicly available project documents, we believe this methodology offers more comprehensive information of Japanese specificities in the development cooperation for health in Vietnam.

In addition, there is a limitation in this study’s methodology. Since this study was based on a cross-sectional analysis, the completeness and the nature of the documentation on projects differed across the sample. Project information such as the project implementation period and project budgets were also assessed predominantly on the basis of the initial project documents. While projects may at times benefit from some adjustment or change in their project paths, some may also suffer a halt during the implementation period, which significantly impacts the overall project scheme. Therefore, such updated information was not reflected in this study.

In terms of capturing the overall Japanese resources in development cooperation for health, there were some limitations of this study. First, we had no systematic way of identifying other possible Japanese interventions except for the major Japanese independent administrative agencies in the fields of health and development. Thus, this study did not cover other potential health projects, especially research projects using public funds that worked in collaboration with Vietnamese institutions. Second, the target projects were collected primarily based on the publicly available information. However, given the different structure of each agency’s website that we accessed, we may have missed some information while navigating each system that may have resulted in a biased collection of data. Third, as only 39 of the 68 projects disclosed their project budgets, our data may have been biased. This data also does not provide a whole picture of Japan’s publicly funded contributions to development cooperation for health in Vietnam. However, this study observed that ODA projects, in particular, large budget schemes such as loan-aid and technical cooperation, disclosed the project budget information. It is possible that they are obligated to adhere to ODA’s transparency policy [7, 61].

Fourth, several Japanese funded projects have strived together with Vietnamese counterparts on specific health focus areas such as infectious diseases and maternal and child health. These specific health focus areas can be captured by using the OECD methodology [62]. However, our study did not employ it. This is because capturing disease related projects cannot explain the focus of Japan’s publicly funded projects for HSS. Based on the data of the current study, future studies can assess the distribution of Japanese projects by health administration level based on the framework of “capacity building,” “infrastructure,” “medical equipment,” and “research and development” to lend insight into the characteristics of Japan’s contribution for development cooperation for health. Last, the results of this study cannot be generalized to Japan’s overall development cooperation for health since this was a cross-sectional study focusing on Japan’s cooperation initiatives with only one country. In addition, the extent to which Japan’s ODA and non-ODA public funds are used would vary from country to country; although there is no publicly available information on Japanese non-ODA public funds used in countries other than Vietnam. Given Japan’s ODA budgets and Japan-Vietnam socio-economic and political relations, there would be more projects in Vietnam utilizing these funds than in other countries.

The analytic approach adopted in this study needs to be developed further to capture a more realistic proportion of each area that the projects worked on. Despite its limitations, the WHO’s six building blocks framework can be utilized with such an arrangement to try to capture efforts on health system strengthening. However, this method needs to be carefully complemented with detailed qualitative information of projects to provide more comprehensive results. Although the assessment framework for health system strengthening needs improvement, this study was the first to assess Japan’s development cooperation for health in a specific recipient country, by including projects funded by both ODA and non-ODA financial resources. In the future, longitudinal studies on Japan’s health cooperation with Vietnam are expected. Further external reviews on Japan’s development cooperation for health in other recipient countries are also necessary for formulating effective cooperation strategies. An assessment of the mobilization of other Japanese resources from the private sector and private philanthropy is also needed in a future study, considering that these resources are expected to drive health cooperation in the global health architecture [63].

Lastly, donor countries should examine international cooperation to strengthen the health system of the target country as some top ODA donor countries do [28, 50, 64]. Objective and systematical reviews of health cooperation based on internationally common assessment frameworks such as the one used in the current study should be promoted. Consequently, the review results should be reflected in the development of recommendations on the cooperation strategy. Based on the current study’s review, priority setting should be strategized and the synergetic effects of various projects employing Japanese resources should be increased to realize efficient and effective development cooperation for health.

## Conclusion

Health is one of the most prioritized areas in Japan’s development cooperation, and the need for health cooperation is increasing globally. With the growing number of project actors within Japan, this research was the first to describe and assess Japan’s publicly funded projects by both ODA and other non-ODA public funds for the development cooperation for health in Vietnam. A number of Japanese funded projects addressed a wide range of health issues across all six building blocks of the health system in Vietnam. However, there is room for improvement in developing coordination and harmonization among Japanese projects that are diversifying. Moreover, establishing a target country-specific mechanism for strategic coordination across Japanese ministries’ schemes is expected for efficient and effective development cooperation for health. While the impact on Vietnam, whose dependence on external funding is low, may be minimal, the impact of insufficient domestic coordination in a donor country on aid-dependent countries could be large in magnitude. Therefore, the importance of coordination across domestic actors of the donor countries can be applied as an important lesson for donor countries, including Japan, in the event that they work in aid-dependent countries.

## Supplementary Information

**Additional file 1: Table S1.** Depicting internet search strategy details.

**Additional file 2: Table S2.** Descriptive details of each project by schemes and their operating organizations and funding ministries, project actors and counterparts, activity components and the corresponding six building blocks, and disclosure of project budget.

**Additional file 3: Table S3.** Categorization of projects by activity components.

**Additional file 4: Table S4.** Proportion of projects by WHO six building blocks and level of health administration, 2016.

## Data Availability

Public access to the databases is open. The datasets used or analyzed during the current study are available from the corresponding author on reasonable request.

## Abbreviations

AMED: Japan Agency for Medical Research and Development
COVID-19: Coronavirus disease 2019
DAC: Development Assistance Committee
DOHA: Direction Office for Healthcare Activities
HSS: Health systems strengthening
JICA: Japan International Cooperation Agency
METI: Ministry of Economy, Trade and Industry
MHLW: Ministry of Health, Labour and Welfare
NCDs: Noncommunicable diseases
OECD: Organisation for Economic Co-operation and Development
ODA: Official Development Assistance
PHC: Primary health care
PPP: Public-Private Partnership scheme
UHC: Universal health coverage
WHO: World Health Organization
